# PIGH deficiency can be associated with severe neurodevelopmental and skeletal manifestations

**DOI:** 10.1111/cge.13877

**Published:** 2020-11-27

**Authors:** Camille Tremblay‐Laganière, Rauan Kaiyrzhanov, Reza Maroofian, Thi Tuyet Mai Nguyen, Kamran Salayev, Ilana T. Chilton, Wendy K. Chung, Jill A. Madden, Chanika Phornphutkul, Pankaj B. Agrawal, Henry Houlden, Philippe M. Campeau

**Affiliations:** ^1^ Department of Pediatrics CHU Sainte‐Justine Montreal QC Canada; ^2^ Department of Neuromuscular Diseases UCL Queen Square Institute of Neurology and The National Hospital for Neurology and Neurosurgery London UK; ^3^ Department of Neurology Azerbaijan Medical University Baku Azerbaijan; ^4^ Departments of Pediatrics Columbia University Irving Medical Center New York NY USA; ^5^ Department of Medicine Columbia University Irving Medical Center New York NY USA; ^6^ Division of Genetics and Genomics Manton Center for Orphan Disease Research, Boston Children's Hospital Boston MA USA; ^7^ Departments of Pediatric and Pathology The Warren Alpert Medical School of Brown University Providence RI USA; ^8^ Division of Newborn Medicine Boston Children's Hospital Boston MA USA

**Keywords:** alkaline phosphatase, delayed myelination, developmental delay, epilepsy, GPI, hypotonia, IGD, iron overload, language delay

## Abstract

Phosphatidylinositol Glycan Anchor Biosynthesis class H (PIGH) is an essential player in the glycosylphosphatidylinositol (GPI) synthesis, an anchor for numerous cell membrane‐bound proteins. PIGH deficiency is a newly described and rare disorder associated with developmental delay, seizures and behavioral difficulties. Herein, we report three new unrelated families with two different bi‐allelic *PIGH* variants, including one new variant p.(Arg163Trp) which seems associated with a more severe phenotype. The common clinical features in all affected individuals are developmental delay/intellectual disability and hypotonia. Variable clinical features include seizures, autism spectrum disorder, apraxia, severe language delay, dysarthria, feeding difficulties, facial dysmorphisms, microcephaly, strabismus, and musculoskeletal anomalies. The two siblings homozygous for the p.(Arg163Trp) variant have severe symptoms including profound psychomotor retardation, intractable seizures, multiple bone fractures, scoliosis, loss of independent ambulation, and delayed myelination on brain MRI. Serum iron levels were significantly elevated in one individual. All tested individuals with PIGH deficiency had normal alkaline phosphatase and CD16, a GPI‐anchored protein (GPI‐AP), was found to be decreased by 60% on granulocytes from one individual. This study expands the PIGH deficiency phenotype range toward the severe end of the spectrum with the identification of a novel pathogenic variant.

## Graphical abstract

GPIs are synthetized by the GPI‐GnT, comprising PIGH, on the cytoplasmic membrane of ER from a phosphatidylinositol and a N‐acetylglucosamine. Once flipped to the luminal side of the ER and further synthesized, GPIs are added to proteins to form GPI‐APs. GPI‐APs are transported to the cytoplasmic membrane via the Golgi. Features of individuals with bi‐allelic *PIGH* variants are presented according to their frequency.

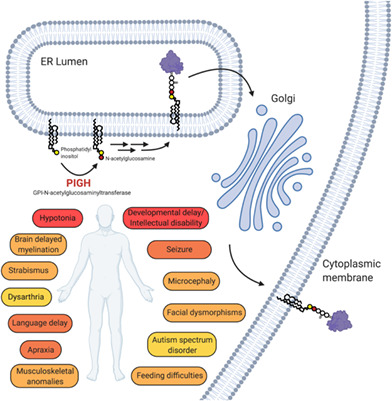

## 1 INTRODUCTION

It is estimated that about 1% of the human proteome is anchored to the cell membrane by a glycosylphosphatidylinositol (GPI) anchor.^1^ GPIs are synthetized initially by the GPI‐N‐acetylglucosaminyltransferase (GPI‐GnT) on the cytoplasmic membrane of endoplasmic reticulum (ER).^2^ GPI‐GnT is a complex comprising PIGA, PIGC, PIGH, PIGP, PIGQ, and PIGY subunits. Absent or abnormal GPI‐anchoring can lead, among other fates, to intracellular degradation or extracellular secretion of the GPI‐anchored proteins (GPI‐APs).^3^ Because of GPI‐Aps' importance in embryogenesis and neurogenesis, disruption of GPI biosynthesis results most commonly in developmental delay/intellectual disability (DD/ID), seizures, dysmorphisms and other nervous system abnormalities such as hypotonia, a group of diseases called inherited GPI deficiency disorders (IGDs).^2^

Phosphatidylinositol Glycan Anchor Biosynthesis class H (PIGH, MIM reference number 618010) is an essential component for GPI‐GnT enzymatic activity in yeast.^4^ There are only three reported cases from two families of individuals with bi‐allelic *PIGH* variants in the literature.^5^, ^6^ Those individuals had DD/ID, seizures, behavioral difficulties, autism spectrum disorder, delayed language, microcephaly, mild dysmorphic features, normal alkaline phosphatase and nonspecific brain MRI findings. Herein, we report three new unrelated families with two different bi‐allelic *PIGH* variants, including one that was never reported before: p.(Arg163Trp).

## 2 RESULTS

**FIGURE 1:**
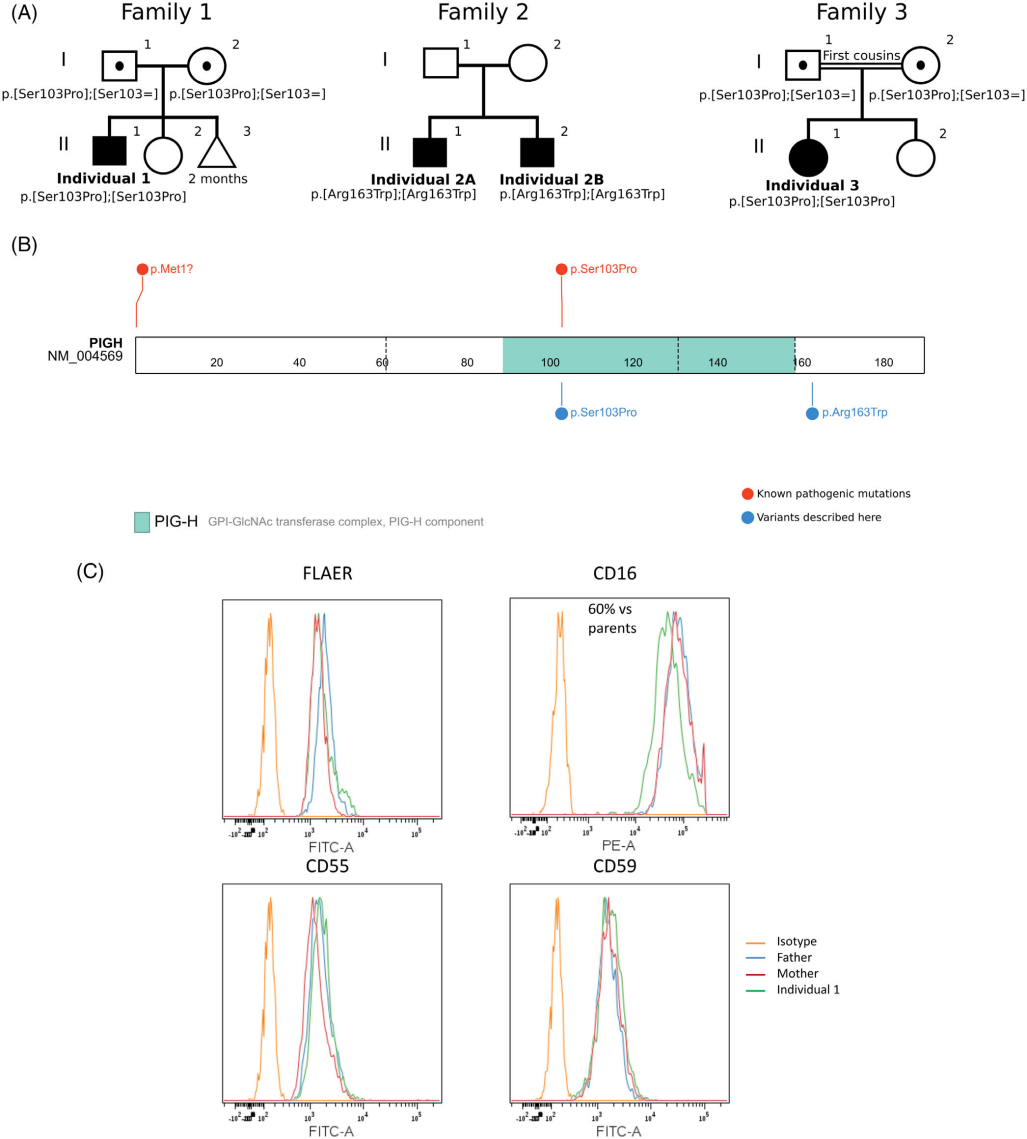
A, Pedigrees and genotypes of families with Bi‐allelic *PIGH* variants. B, PIGH protein structure and location of variants. Figure from ProteinPaint. Variants from affected individuals described here are in blue. Previously published pathogenic variants are in red.^5^, ^6^ C, FACS from blood cells of one affected individual GPI‐AP surface levels (FLAER, CD16, CD55, and CD59) on granulocytes for individual 1 [Colour figure can be viewed at wileyonlinelibrary.com]

The core phenotype in this case series is DD/ID combined with hypotonia. Family pedigrees are presented in Figure 1A. Table 1 compares features from previously reported individuals to individuals described herein.

**TABLE 1:** Phenotypic features of individuals with biallelic inactivating variants in *PIGH*

Individual	1	2A	2B	3	Total	Previously published
Hypotonia	+	+	+	+	4/4	1/3
DD/ID	+	+++	+++	+	4/4	3/3
Language delay	+++	+++	+++	−	3/4	2/3
Epilepsy/seizures	−	++	+	+	3/4	3/3
Apraxia	+	+++	+++	−	3/4	0/3
Feeding difficulties	−	+	+	−	2/4	0/3
Facial dysmorphisms	−	+	+	−	2/4	2/3
Microcephaly	−	+	+	−	2/4	2/3
Strabismus	−	+	+	−	2/4	0/3
Musculoskeletal anomalies	−	++	+	−	2/4	0/3
Autism spectrum disorder or behavioral difficulties	++	−	−	−	1/4	3/3
Dysarthria	+	−	−	−	1/4	0/3
Delayed myelination at brain MRI	N/A	+	+	−	2/4	0/3
Normal serum alkaline phosphatase	N/A	+	N/A	+	2/4	3/3
Elevated serum iron	N/A	N/A	N/A	+	1/4	0/3

*Note:* N/A = Not Available. DD/ID = Developmental Disability and/or Intellectual Disability. + = mild, ++ = moderate, +++ = severe, − = absent.

The affected individual in Family 1 is a 4‐year‐old boy with an unremarkable antenatal and neonatal history. He has an asymptomatic sister and was born to non‐consanguineous parents of Indian origin. He did not have a motor delay but language acquisition was significantly impaired. No speech or babbling were noted at 18 months old, and he is currently nonverbal. There was no history of seizures. He has a confirmed autism spectrum disorder. His neuromuscular examination at 18 months old revealed apraxia, dysarthria, and hypotonia. Height was 84.5 cm (80th percentile), weight was 11.3 kg (37th percentile) and head circumference was 48.5 cm (72nd percentile). The rest of the physical examination was unremarkable. CGH microarray was normal. Whole exome sequencing showed KIAA2022 (p.T1254N, c.C3761A) and BCOR (p.L532S, c.T1595C) maternally inherited VUSs (variants of uncertain significance).

Family 2 presented with two affected siblings born to non‐consanguineous parents of Guatemalan descent. The proband is a 20‐year‐old male, who was born at 38 weeks of gestation to a mother with insulin‐controlled gestational diabetes. He was remarkably underweight at the seventh percentile at birth and fourth percentile at 18 years old. Severe DD/ID and poorly controlled seizures were the predominant neurological features. He began having focal seizures on the 10th day of life with mostly nocturnal episodes lasting for 1‐2 minutes or longer. EEG at 18 years old was within the broad limits of normal in the awake state but excessive beta waves were present throughout the tracing. Trials of phenobarbital, phenytoin, carbamazepine, valproic acid, and zonisamide did not permit to achieve seizure control. He is currently on a combination of topiramate, levetiracetam, and clonazepam and continues to have 2‐3 seizures a month. He had a history of multiple bone fractures due to osteopenia and a scoliosis, which was surgically corrected by posterior fusion from T2 to the pelvis. His independent ambulation was impaired, requiring a wheelchair, and he was nonverbal. Upon examination, apraxia, microcephaly (head circumference below first percentile) and significant overbite were present. Feeding was through a gastrostomy tube and he suffered constipation. He was found to have esotropia, hypotonia, and upper limbs dystonia coupled with flexible joints and lumbar lordosis. The rest of the physical examination was unremarkable. Renal ultrasound and plasma alkaline phosphatase levels were normal. Brain MRI at 2 years old revealed delayed myelination and diffuse abnormal signal in the right temporal lobe. His affected brother is 7 years old. He was also born full term, and the pregnancy was complicated by insulin‐controlled gestational diabetes. The neonatal period was unremarkable. He had severe DD/ID with early‐onset seizures like his brother. Focal seizures began at 3 months old. Valproic acid and diazepam were unsuccessful. He had been seizure free for several years with topiramate, levetiracetam and clonazepam. EEG at 4 years old demonstrated interictal spikes with a slow background. He had constipation, and his diet is restricted to pureed foods. The physical exam showed a myopathic facies with anteverted nares, esotropia and microcephaly (head circumference below first percentile). He also had flexible joints with scoliosis, hypotonia, dystonia and apraxia. He is non‐verbal and wheelchair bound. MRI showed delayed myelination at 1 year old. Hearing exam, amino acids, urine organic acids, acylcarnitine, and very long‐chain fatty acid levels were normal for both siblings.

The proband in Family 3 is a 4‐year‐old girl, who is the first child of a consanguineous couple of Azerbaijani origin. She was born full‐term after an uneventful pregnancy but suffered asphyxia at birth. Her neonatal physical measurements were normal. She had febrile seizures at 7 months old and mild motor delay. From one and half year old, she developed afebrile seizures including absence and generalized tonic seizures. Her EEG showed generalized spike‐and‐wave complexes, and the seizures failed to respond to various antiepileptic medications including topiramate, ethosuximide, phenobarbital, and levetiracetam. Only valproic acid 600 mg/day was effective, and she is currently seizure‐free on this medication with a normalized EEG. She is verbal, speaks in full sentences, although there is a very mild cognitive delay with no autistic features and no behavioral abnormalities. Except for hypotonia, her physical examination along with her brain MRI was unremarkable. She has normal head circumference. Plasma alkaline phosphatase levels were within a normal range (342.0 U/L) and plasma iron was elevated (34.3 μmol/L) with normal ferritin (17.5 ng/mL). She did not receive iron supplementation. She has one sibling, who is currently unaffected.

The four affected individuals from three families described here were homozygous for one *PIGH* missense variants (p.(Ser103Pro) for Families 1 and 3, and p.(Arg163Trp) for Family 2). Figure 1B shows localization of these variants across the protein in relation with the GPI‐GIcNAc transferase complex domain. The variant p.(Ser103Pro) was previously reported.^6^ One additional variant p.(Met1Leu) was previously published and resulted in an alternative in‐frame start in exon 2 (Met63).^5^ The variant p.(Ser103Pro) was predicted to be a VUS by ACMG classification and has a CADD score of 27.7 (See Table S1). Pathogenicity prediction tools from Varsome were divided in their predictions (see Table S1). The variant p.(Arg163Trp) was predicted to be a VUS by ACMG classification and has a CAAD score of 32. More tools predicted the variant to be pathogenic than not. Both variants occur in residues well conserved across vertebrates (See Figure S1).^7^

A blood sample was obtained from Individual 1, and granulocytes were tested for GPI‐AP levels and compared to those of his unaffected heterozygous parents (Figure 1C). FLAER, CD55 and CD59 levels were unchanged but CD16 levels were decreased by 60%.

## 3 DISCUSSION

Previously reported individuals with PIGH deficiency had cognitive performance allowing attending mainstream school with support.^5^, ^6^ In our study, three individuals were nonverbal with moderate or severe DD/ID. Autism spectrum disorder/behavioral difficulties, which was a predominant feature in the previously reported cases, was only present in one individual from this series. Brain MRI in a previous study showed subtle nonspecific findings and PET revealed increased glucose uptake in the temporal regions in one individual.^5^ Herein, we found delayed myelination in two siblings from one family and diffuse abnormal signal in the right temporal lobe in one, whereas in others brain imaging appeared normal. Delayed myelination was also observed in individuals with PIGA and PIGQ deficiency.^2^, ^8^ Esotropia, dysarthria, dystonia, and spinal deformities coupled with osteopenia leading to multiple fractures, loss of independent ambulation, and elevated serum iron levels are among the previously unreported symptoms associated with PIGH deficiency in our series. Systemic iron overload was previously described in one family with neurodegeneration, dermatosis including ichthyosis and pathogenic variants in *PIGA*, another GPI‐GnT component.^9^ The author's hypothesis was that some proteins involved in iron homeostasis, notably hemojuvelin, are GPI‐anchored and disruption of their anchor could result in a dysregulation leading to progressive systemic iron overload. One individual from a previous study had dry skin suspected to be ichthyosis but serum iron or ferritin were not measured—ichthyosis is also seen with PIGL deficiency.^2^, ^5^ Craniofacial dysmorphisms, strabismus, and musculoskeletal anomalies, namely scoliosis and osteopenia/osteoporosis are often seen in other IGDs. In contrast with other IGDs, all tested individuals with PIGH deficiency had normal alkaline phosphatase which has a GPI‐anchor form.^2^, ^5^

Drawing genotype‐phenotype correlations in PIGH deficiency is difficult due to the limited number of reported cases. However, all individuals with p.(Ser103Pro) have hypotonia, mild to moderate DD/ID with variable language impairment and seizures. The individuals from Family 2 (homozygous p.(Arg163Trp)) in our report have the most severe phenotype compared to all other affected individuals, including those from the literature. Surprisingly, Arg163, in contrast to Ser103, is outside the GPI‐GIcNAc transferase complex and is closer to the PIGH C‐terminal end (Figure 1B). Nevertheless, the variant could affect the protein's interactions, localization, stability, or indirectly affect its enzymatic activity. This outlines the need for further characterization of PIGH physiologic role and functional assessment of the impact of different variant on the protein.

The two previous reports of *PIGH* bi‐allelic variants demonstrated GPI deficiencies with their variant. A previously described individual homozygous for p.(Ser103Pro) was found to have decreased CD55 level as well as a 51% decrease in CD16 on granulocytes compared to asymptomatic carrier parents.^6^ In the present series, we showed a 60% decrease in surface CD16 but normal FLAER, CD55 and CD59 levels on homozygous p.(Ser103Pro) granulocytes compared to both unaffected heterozygous parents.

In summary, our report helps to better delineate the phenotypic features of *PIGH* deficiency, reveals previously unreported disease‐associated symptoms and expands the phenotype including severe neurodevelopmental manifestations.

## 4 MATERIAL AND METHODS

### 4.1 Identification of affected individuals

Individuals were recruited by contact with their clinicians or through GeneMatcher. Informed written consent was obtained. Ethical approval was granted by CHU Sainte‐Justine Research Ethics Board. Family 2 was consented to the Manton Center for Orphan Disease Research Gene Discovery Core protocol (IRB: 10‐02‐0053).

### 4.2 Analysis of *PIGH* variants

*PIGH* variant characteristics were extracted from Varsome.^10^ CADD scores were calculated on https://cadd.gs.washington.edu.^11^ *PIGH* variants were shown in ProteinPaint from St. Jude Children's Research Hospital PeCan Data Portal.^12^

### 4.3 Blood fluorescence‐activated cell sorting

Blood samples were incubated on ice with GPI‐AP markers: PE‐CD16 (BioLegend), FITC‐ CD55, FITC‐CD59 (BD PharMingen), and FLAER‐Alexa 448 (Cedarlane). Samples were washed using a BD FACSCanto II system (BD Biosciences). Analysis was carried out with Cytobank software.

## CONFLICT OF INTEREST

No conflict of interest to declare.

## AUTHOR CONTRIBUTIONS

CTL and RK wrote the manuscript. TTMN performed experiments. PMC designed the study and interpreted the data. RK, RM, KS, ITC, WKC, JM, CP, PBA and HH provided clinical information and material. WKC contributed clinical and genomic data and provided critical review of the manuscript.

### PEER REVIEW

The peer review history for this article is available at https://publons.com/publon/10.1111/cge.13877.

## Supporting information


**Figure S1** Conservation of the Two Affected Residues across vertebrates Multiple alignment from USCS genome browser^12^

**Table S1** List of the *PIGH* Variants Identified in the Subjects Included in the Study

## Data Availability

Data sharing is not applicable to this article as no new data were created or analyzed in this study.
