# Supplementary material for: PIGH deficiency can be associated with severe neurodevelopmental and skeletal manifestations
Source: Clin Genet. 2020 Nov 27;99(2):313–7. doi: 10.1111/cge.13877 (PMC7839508; doi:10.1111/cge.13877)
Supplement: Supplementary file 1 — Figure S1 Conservation of the Two Affected Residues across vertebrates Multiple alignment from USCS genome browser12 [file CGE-99-313-s001.pdf]

Figure S1

					Ser103				
Human	Q	M	T	S	S	Y	A	S	G
Rhesus	Q	M	T	S	S	Y	A	S	G
Mouse	Q	M	T	S	S	Y	A	S	G
Dog	Q	M	T	S	S	Y	A	S	G
Elephant	N	L	T	P	S	Y	T	S	A
Chicken	Q	V	T	S	S	Y	A	S	G
Zebrafish	Q	L	S	S	S	Y	A	S	G

					Arg163				
Human	S	A	K	P	R	L	D	C	L
Rhesus	S	A	K	P	R	L	D	C	L
Mouse	S	A	K	P	R	L	D	C	L
Dog	S	A	K	P	R	L	D	C	L
Elephant	S	A	K	P	R	L	D	C	L
Chicken	S	A	K	P	R	L	D	C	L
Zebrafish	S	S	Q	P	R	L	D	C	L

Previously published

					Met1				
Human	-	-	-	-	M	E	D	E	R
Rhesus	-	-	-	-	M	E	D	E	R
Mouse	-	-	-	-	M	E	D	E	K
Dog	-	-	-	-	M	E	D	E	R
Elephant	-	-	-	-	M	K	D	E	R
Chicken	-	-	-	-	M	E	-	-	R
Zebrafish	-	-	-	-	M	A	D	-	-
