# Supplementary material for: PIGH deficiency can be associated with severe neurodevelopmental and skeletal manifestations
Source: Clin Genet. 2020 Nov 27;99(2):313–7. doi: 10.1111/cge.13877 (PMC7839508; doi:10.1111/cge.13877)
Supplement: Supplementary file 2 — Table S1 List of the PIGH Variants Identified in the Subjects Included in the Study [file CGE-99-313-s002.docx]

| **Table S1. List of the *PIGH* Variants Identified in the Subjects Included in the Study** | | |  |  |  |  |  |  |
| --- | --- | --- | --- | --- | --- | --- | --- | --- |
| **Family** | **Genomic Variant (hg 19)** | **DNA Variant (RefSeq: NM_004569)** | **Protein Variant** | **Inheritance** | **gnomAD Minor Allele Frequency** | **Pathogenicity ACMG classification** | **Pathogenicity CADD score (GRCh37-v1.6)** | **Pathogenicity from other tools via Varsome (mostly from dbNSFP)** |
| **1 and 3** | chr14:g.68060543A>G | c.307T>C | p.Ser103Pro | homozygous | 0.0000159, highest 0.0000352 (European Non-Finnish population) | VUS | 27.7 | Pathogenic predictions from DANN, EIGEN, FATHMM-MKL, MutationAssessor, MutationTaster and SIFT. Benign predictions from DEOGEN2, M-CAP, MVP, PrimateAI and REVEL |
| **2** | chr14:g.68056877G>A | c.487C>T | p.Arg163Trp | homozygous | 0.0000257, highest 0.0000885 (East Asian population) | VUS | 32.0 | Pathogenic predictions from DANN, EIGEN, PrimateAI, FATHMM-MKL, MutationTaster, SIFT, MutationAssessor and DEOGEN2. Benign predictions from M-CAP, MVP and REVEL |
| **Previously published** | chr14:g.68066920T>G | c.1A>C | p.Met1Leu | homozygous | N/A | Likely Pathogenic | 25.1 | Pathogenic predictions from FATHMM-MKL, M-CAP and MutationTaster. Benign predictions from DANN, DEOGEN2, EIGEN, MVP and REVEL |
